# Lower risk of peripheral venous catheter-related bloodstream infection by hand insertion

**DOI:** 10.1186/s13756-022-01117-8

**Published:** 2022-06-03

**Authors:** Niccolò Buetti, Mohamed Abbas, Didier Pittet, Marie-Noëlle Chraiti, Valérie Sauvan, Marlieke E. A. De Kraker, Matthieu Boisson, Daniel Teixeira, Walter Zingg, Stephan Harbarth

**Affiliations:** 1 Infection Control Program and WHO Collaborating Centre on Patient Safety, University of Geneva Hospitals and Faculty of Medicine, Service PCI, Rue Gabrielle-Perret-Gentil 4, 1205 Geneva, Switzerland; 2 UMR 1137, IAME, INSERM, Université de Paris, 75018 Paris, France; 3 MRC Centre for Global Infectious Disease Analysis, Imperial College London, London, UK; 4 Department of Infectious Diseases and Hospital Epidemiology, University Hospital Zurich, Zurich, Switzerland

**Keywords:** Peripheral venous catheters, Bloodstream infections, Catheter, Catheter-infection, Insertion site

## Abstract

**Introduction:**

Little is known about the bloodstream infection (BSI) risk associated with short-term peripheral venous catheters (PVCs) and no large study investigated the insertion site-related risk for PVC-BSI.

**Methods:**

We performed a cohort study at the University of Geneva Hospitals using the prospective hospital-wide BSI surveillance database. We analyzed the association between insertion site and risk of PVC-BSI on the upper extremity using univariable and multivariable marginal Cox models.

**Results:**

Between 2016 and 2020, utilization of 403′206 peripheral venous catheters were prospectively recorded in a 2000-bed hospital consortium with ten sites. Twenty-seven percent of PVC (n = 109′686) were inserted in the hand. After adjustment for confounding factors, hand insertion was associated with a decreased PVC-BSI risk (adjusted hazard ratio [HR] 0.42, 95% CI 0.18–0.98, *p* = 0.046) compared to more proximal insertion sites. In a sensitivity analysis for PVCs with ≥ 3 days of dwell time, we confirmed a decreased PVC-BSI risk after hand insertion (HR 0.37, 95% CI 0.15–0.93, *p* = 0.035).

**Conclusion:**

Hand insertion should be considered for reducing PVC infections, especially for catheters with an expected dwell time of more than 2 days.

**Supplementary Information:**

The online version contains supplementary material available at 10.1186/s13756-022-01117-8.

## Introduction

Short-term peripheral venous catheters (PVCs) are frequently used in hospitalized patients [1]. Several PVC-related complications have been reported, such as hematoma, phlebitis, extravasation and bruising [2]. Interestingly, little is known about the bloodstream infection (BSI) risk associated with PVCs [3]. A recent systematic review suggested that this risk may be underestimated [3].

Conventional wisdom dictates that the distal extremity should be cannulated first and proximal sites should be saved for subsequent cannulation. Small studies focusing on specific microorganisms (i.e., *Staphylococcus aureus*) showed that PVCs inserted in the hand may decrease the risk of PVC-BSI [4]. However, no large study investigated the insertion site-related risk for PVC-BSI. Our hypothesis was that PVC inserted in the hand is associated with a lower infectious risk due to (i) the decreased risk of catheter dislodgement and kinking, thus leading to subsequent dressing disruption and (ii) the ease of monitoring (i.e., observation of correct placement and/or local signs of infection). The objective of this study was to investigate the association between anatomical PVC insertion site and risk of BSI using a large prospective database.

## Material and methods

### Setting, patients and catheters

This cohort study was performed at the University of Geneva Hospitals (HUG), the largest tertiary care center network in Switzerland with ten sites (5 rehabilitation and/or palliative care sites, 1 acute-care site, 1 geriatrics site, 1 pediatrics site, 1 gynecology-obstetrics site, 1 psychiatry site) comprising approximately 2000 beds and 60′000 hospital admissions per year.

We included all hospitalized patients (adult and children) with at least one PVC insertion on the upper extremity between 1^st^ January 2016 and 29th February 2020. The types of PVCs used were not different between the sites. During the study period, PVC with secured injection site were used. Outpatients were excluded. Since the incidence of PVC-BSI inserted in the lower extremity (i.e., feet or legs) is different from upper extremity [5], we excluded all PVC inserted in the lower extremities.

### Data sources and variables collected

Ward type, patient data (age, gender, dates of hospitalization), and PVC data (insertion site, duration of catheter maintenance) were collected from the electronic health record system at HUG. PVC-BSI data (date of onset, pathogen) were collected from the hospital-wide BSI surveillance database, which has been completed prospectively by the Infection Control Program of HUG for over 25 years.

### Definitions

PVC-BSI was defined as a positive blood culture occurring from catheter insertion until 48 h after catheter removal, with the same microorganism as isolated from a positive superficial culture from pus from the insertion site or a quantitative PVC tip culture ≥ 10^3^ CFU/ml (or semi-quantitative CVC culture > 15 colony forming unit) according to the European Centre for Disease Prevention and Control (ECDC) [6]. Alternatively, a positive blood culture occurring from day of insertion until 48 h after catheter removal, the resolution of symptoms in 48 h after catheter removal and the absence of any other infectious focus was included as PVC-BSI as well. Typical skin contaminants were included only if the patient had at least one sign or symptom (chills, hypotension or fever [> 38 °C]) of infection and at least two positive blood cultures from two separate blood samples within 48 h. Typical skin contaminants were coagulase–negative staphylococci (CoNS), *Corynebacterium* spp, *Bacillus* spp, *Micrococcus* spp, and *Propionibacterium* spp. We investigated the risk of BSI for PVC inserted in the upper extremity and defined a binary variable for the insertion site (hand vs. other sites).

### Infection control procedures

Institutional guidelines/policy for PVC insertion were the following: (1) the site of insertion was left to the discretion of the nurse or doctor caring for the patient; (2) alcohol-containing 2% chlorhexidine-gluconate was used for skin antisepsis at catheter insertion and during dressing changes; (3) semipermeable transparent dressings were used at all insertion sites and were changed when clinically indicated. Soiled, leaking or wet dressings were immediately changed. The insertion site was inspected daily. PVCs were routinely replaced every 96 h, except for the period from April 2018 to October 2019, during which PVCs were replaced when clinically indicated only [7].

### Statistical analysis

Characteristics of patients and catheters were described as counts (percent) or medians (interquartile range [IQR]) for categorical and continuous variables, respectively. We performed group comparisons between the different insertion sites using the Chi squared test, Fisher’s exact test and the Wilcoxon test for categorical and continuous variables, as appropriate.

We used a marginal Cox regression model for clustered data to take into account clustering of multiple PVCs per patient, using a robust sandwich covariate estimate and the censored nature of the data. Catheter-days were censored at catheter removal, with a maximum follow-up of 30 days. Hazard ratios (HRs) for PVC-BSI were evaluated by univariable and multivariable analysis. A HR greater than one indicates an increased risk of PVC-BSI. The main variable of interest “insertion site” (i.e., hand vs. other sites) was forced into our multivariable models, and other covariates, which are thought to influence the risk of PVC-BSI, were used as adjustment factors (i.e., age, gender, time from hospital admission to PVC insertion). Since catheterization duration changed according to insertion sites (i.e., hand vs. other sites) and during the study period, we performed sensitivity analyses on subgroups with a different duration of PVC maintenance (i.e., ≥ 3 days or < 3 days, ≥ 4 days or < 4 days; < 5 days, < 6 days, < 7 days, < 8 days and < 9 days). Moreover, we performed an explanatory analysis including only PVC inserted in the hand and forearm (i.e., both insertion sites with lower risk of kinking or dislodgement) in PVC with catheter maintenance ≥ 3 days. The proportionality of hazard risks for the insertion site was tested using Martingale residuals. Tests were two-tailed, with *p* < 0.05 being considered significant. All analyses were performed using SAS (version 9.4; SAS Institute, Cary, NC).

### Ethical considerations

The PVC-BSI surveillance is part of a mandatory indicator surveillance at HUG, and thus, is considered quality assurance; institutional review board approval was not required.

## Results

**Fig. 1:**
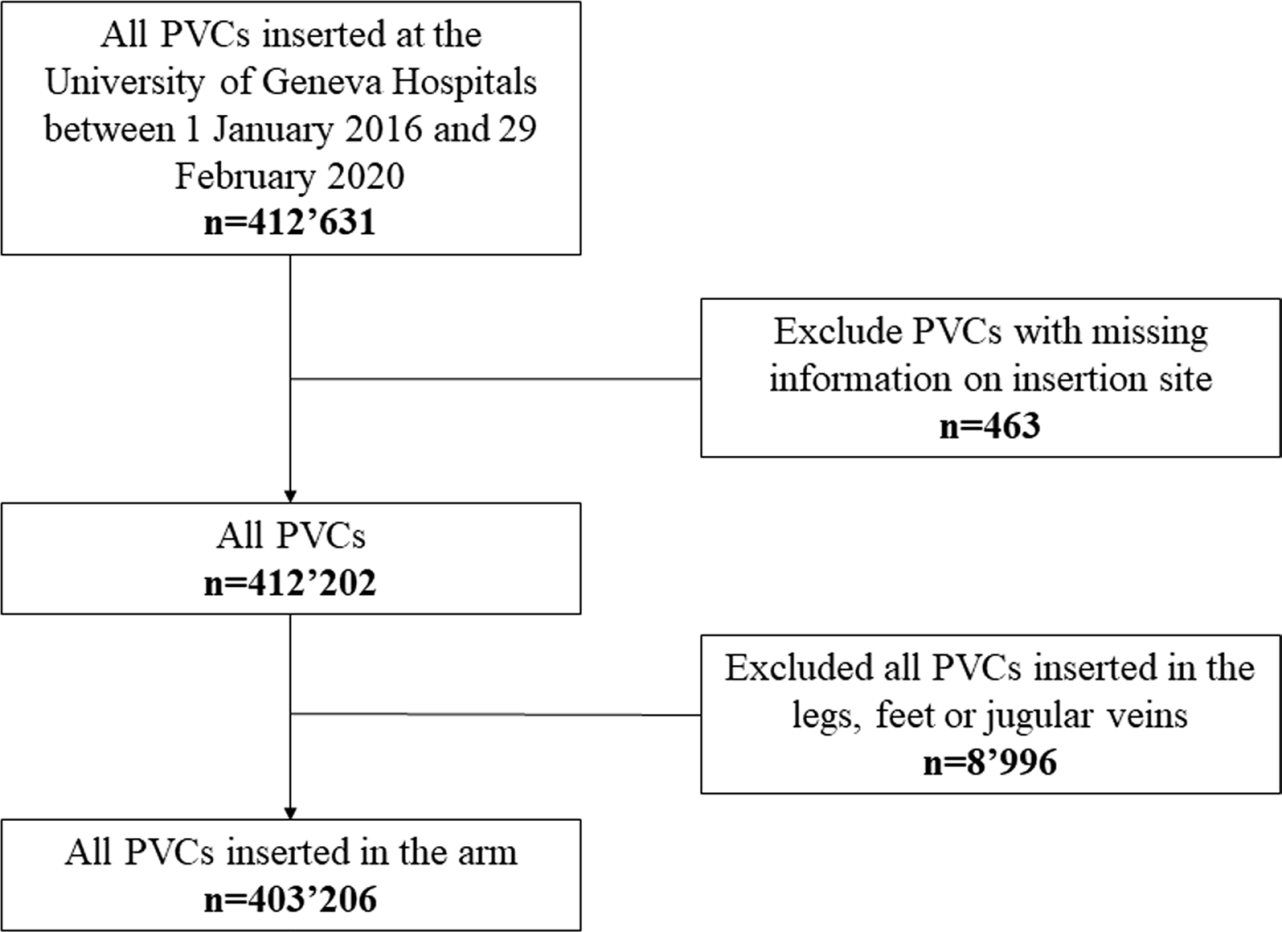
Flow-chart. PVC, Peripheral venous catheter

A total of 403′206 upper extremity PVCs with documented insertion site were included (Fig. 1). There were 73′325 female patients (53.9%), and the median age was 49 years (IQR 32–69, Additional file 1: Table S1). The mean number of PVCs per patient stay was 1.7 (SD 1.59).

The median time from hospital admission to catheter insertion was 1 day (IQR 1–4), and 109′686 (27.2%) PVCs were inserted in the hand. Patients with insertion in the hand were younger compared to other patients (42 years [IQR 28–58] vs. 52 years [IQR 33–72], *p* < 0.01). Catheters inserted at other sites had an increased median dwell time compared to hand inserted PVCs (3 vs. 2 days, *p* < 0.01, Table 1). During the study period, 61 PVC-BSIs were documented, 6 originating from PVCs in the hand, and 55 from PVCs at other insertion sites. The assumption of proportional risks for the variable insertion sites was respected.

**Table 1:** Hand versus other insertion sites

	Other sites	Hand	*p* value
Gender*, female (%)	58,301 (54.3)	15,024 (52.6)	< 0.01
Age*, median [IQR]	52 [33; 72]	42 [28; 58]	< 0.01
ICU (%)	7237 (2.5)	1915 (1.7)	< 0.01
Time to catheter insertion, median [IQR]	1 [1; 5]	1 [1; 2]	< 0.01
Catheter days, median [IQR]	3 [2; 4]	2 [1; 4]	< 0.01
Catheter ≥ 3 days (%)	155,932 (53.1)	48,084 (43.8)	< 0.01
Insertion outside of the hospital (%)	15,044 (5.1)	17,101 (15.6)	< 0.01
PVC-related BSI (%)	55 (0)	6 (0)	< 0.01

IQR, interquartile range; ICU, intensive care unit; PVC, peripheral venous catheter; BSI, bloodstream infection. *135′969 patients

**Fig. 2:**
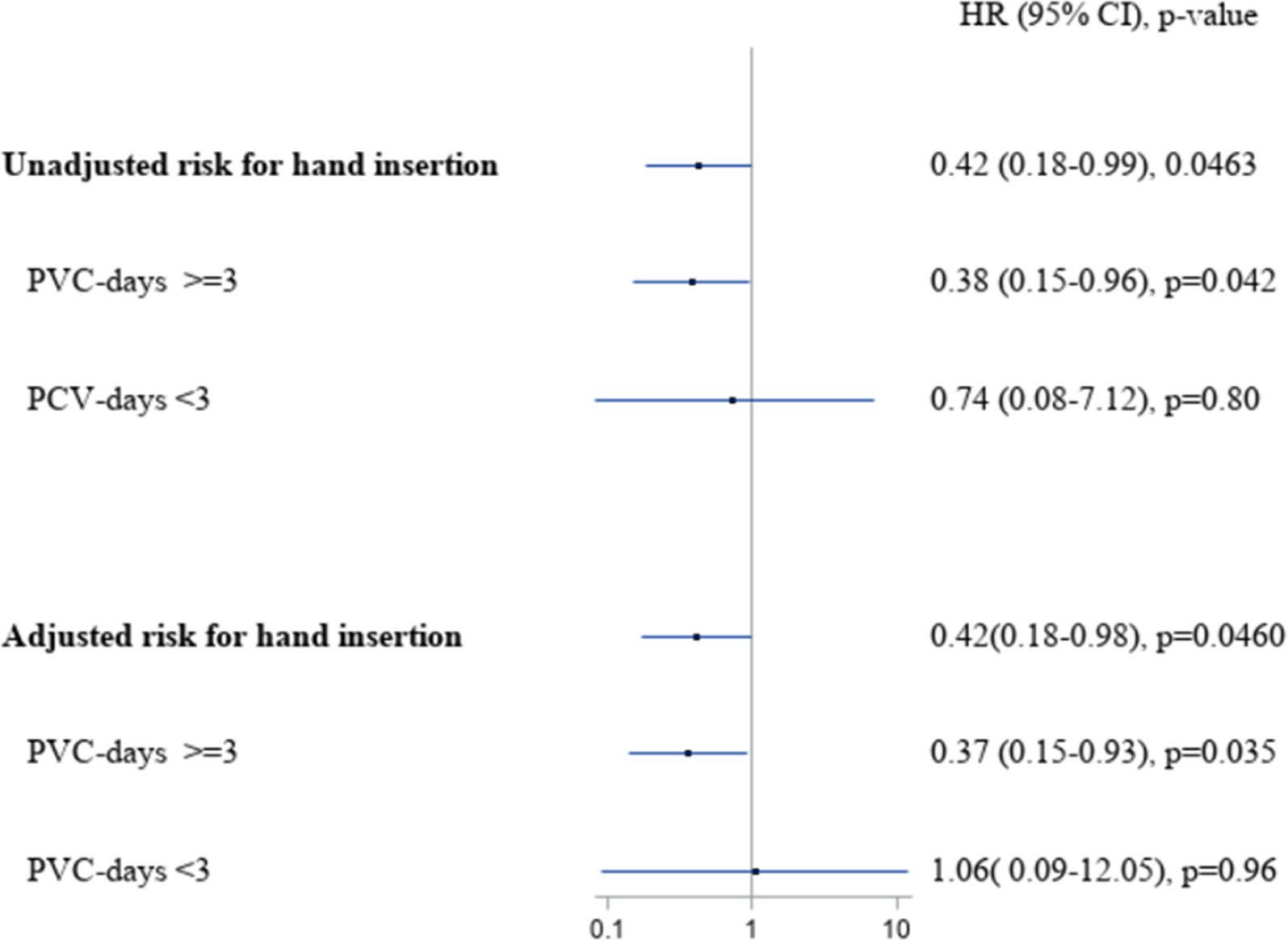
Unadjusted and adjusted PVC-BSI hazard ratio for hand insertion versus other upper extremity sites stratified by catheter duration. HR, hazard risk; CI, confidence interval; PVC, peripheral venous catheter. Adjustment variables: Sex, age and time admission—PVC insertion

After univariable marginal Cox modelling, hand insertion was associated with a decreased risk for PVC-BSI compared to the other sites (HR 0.42, 95% CI 0.18–0.99, *p* = 0.046, Fig. 2 and Additional file 1: Table S2). Interestingly, female patients had a reduced risk of PVC-BSI compared to male patients (HR 0.36, 95% CI 0.2–0.64, *p* = 0.0006, Additional file 1: Table S2). After adjustment for gender, age and time from admission to catheter placement, hand insertion correlated with a decreased PVC-BSI risk (adjusted HR 0.42, 95% CI 0.18–0.98, *p* = 0.046, Fig. 2 and Additional file 1: Table S3). Again, female patients had a decreased risk of PVC-BSI compared to male patients (adjusted HR 0.36, 95% CI 0.20–0.65, *p* = 0.0007, Additional file 1: Table S3).

In a sensitivity analysis for PVCs with ≥ 3 days of dwell time, we confirmed a decreased PVC-BSI risk after hand insertion (HR 0.37, 95% CI 0.15–0.93, *p* = 0.035); whereas for PVC < 3 days, a similar PVC-BSI risk between hand and other sites was observed (HR 1.06, 95% CI 0.093–12.05, *p* = 0.96). In a sensitivity analysis including only PVCs with ≥ 4 days of dwell time, we confirmed a decreased PVC-BSI after hand insertion (HR 0.41, 95% CI 0.16–1.02, *p* = 0.056). Similar results were observed for PVCs for longer catheter durations (Additional file 1: Table S4). An explanatory analysis including only hand and forearm inserted PVCs for at least 3 days (157′572 PVCs), showed similar results for hand insertion (HR 0.43, 95% CI 0.167–1.0, *p* = 0.077). In particular, PVC-reinsertions (vs. first insertions) showed the lowest risk for hand insertion (HR 0.349, 95% CI 0.12–0.99, *p* = 0.047).

Coagulase negative staphylococci were the most frequently observed microorganisms (Additional file 1: Table S5). No difference in microorganism distribution between hand and other sites was observed.

## Discussion

Using a large prospective database including more than 400′000 PVCs, we showed that PVCs inserted in the hand were associated with a significantly decreased daily risk of BSI compared to PVCs inserted more proximally.

Probably due to their low incidence, PVC-related infections are rarely investigated. High quality studies mostly described other more common complications such phlebitis, extravasation, hematoma or bruising [2, 8]. A recent systematic review illustrated that antecubital fossa veins were associated with lower phlebitis rates, while hand veins showed the highest risk for phlebitis [9]. However, to our knowledge, no study investigated the association between PVC insertion site and catheter-related BSI, which may cause increased length of stay and mortality [10]. Using a large hospital network with consistent catheter care, we showed that PVC inserted in the hand had a decreased risk for bloodstream infection. Our sensitivity analyses found that the PVC-BSI risk was especially decreased after two or three days of dwell time.

Our cohort did not allow an in-depth pathophysiological explanation of these results: it is conceivable that daily exit-site inspection was better for hand-PVCs compared to other sites. Moreover, hand insertions were less prone to dislodgement and kinking compared to sites over joints (e.g., antecubital fossa or wrist). This may influence catheter-dressing disruptions, which represents a well-known risk factor for central venous catheter infections [11]. However, an explanatory analysis including hand and forearm inserted PVCs (i.e., both PVC insertions with low risk of dislodgement or kinking) showed again a lower risk for hand insertions, thus mitigating this hypothesis.

Infectious Disease of America Society (IDSA) guidelines [12] and the Center Disease Control [13] recommend the use of the upper extremities for PVC with no specification of preferred anatomical insertion site. In light of our results and in order to preserve proximal veins for further cannulation, we would prefer to recommend hand insertions for PVCs, especially if longer dwell-times are to be expected.

Several limitations should be acknowledged. First, we performed an observational study with limited access to clinical data (e.g., comorbidities, severity of disease, experience of the inserter) and the insertion sites were not randomized; thus, confounding by indication could be present. Second, our multivariable Cox models were adjusted only for a limited number of available covariates. Moreover, due to the low incidence of infection in PVC, few PVC-BSIs were observed in the hand insertion group. In this context, results of multivariable analyses should be interpreted with caution. Furthermore, our models did not take into account the reason for PVC removal (i.e., phlebitis), which may lead to PVC removal at an early stage.

## Conclusion

Using a large prospective database, we showed a decreased daily risk for infection after PVC insertion in the hand versus other upper extremity sites. Hand insertion should be considered for reducing PVC infections, especially for catheters with an expected dwell time of more than two days.

## Supplementary Information

**Additional file 1: Table S1**. Patients with peripheral catheters in the upper extremity. **Table S2**. Univariable Cox model for PVC-related BSI. **Table S3**. Multivariable marginal Cox model for PVC-related BSI. **Table S4**. Univariable Cox models for PVC-related BSI stratified by different PVC duration. **Table S5**. Microorganism distribution according to the insertion site.

## Data Availability

The datasets generated during and/or analyzed during the current study are available from the corresponding author on reasonable request.

## Abbreviations

BSI: Bloodstream infection
CoNS: Coagulase–negative staphylococci
ECDC: European Centre for Disease Prevention and Control
HR: Hazard ratio
HUG: University of Geneva Hospitals
IDSA: Infectious Disease of America Society
IQR: Interquartile range
PVC: Short-term peripheral venous catheters

## References

[1] Alexandrou E, Ray-Barruel G, Carr PJ, Frost SA, Inwood S, Higgins N (2018). Use of short peripheral intravenous catheters: characteristics, management, and outcomes worldwide. J Hosp Med.

[2] Hadaway L (2012). Short peripheral intravenous catheters and infections. J Infus Nurs.

[3] Mermel LA (2017). Short-term peripheral venous catheter-related bloodstream infections: a systematic review. Clin Infect Dis.

[4] Trinh TT, Chan PA, Edwards O, Hollenbeck B, Huang B, Burdick N (2011). Peripheral venous catheter-related Staphylococcus aureus bacteremia. Infect Control Hosp Epidemiol.

[5] Salgueiro-Oliveira A, Parreira P, Veiga P (2012). Incidence of phlebitis in patients with peripheral intravenous catheters: the influence of some risk factors. Austral J Advanc Nurs.

[6] ECDC. Surveillance of healthcare-associated infections and prevention indicators in European intensive care units. Available from: https://www.ecdc.europa.eu/sites/default/files/documents/HAI-Net-ICU-protocol-v2.2_0.pdf. 2017.

[7] Buetti N, Abbas M, Pittet D, de Kraker MEA, Teixeira D, Chraiti MN (2021). Comparison of Routine Replacement With Clinically Indicated Replacement of Peripheral Intravenous Catheters. JAMA Intern Med..

[8] Webster J, Osborne S, Rickard CM, Marsh N. Clinically-indicated replacement versus routine replacement of peripheral venous catheters. Cochrane Database Syst Rev. 2019;1:CD007798. 10.1002/14651858.CD007798.pub5 PMC6353131 30671926

[9] Comparcini D, Simonetti V, Blot S, Tomietto M, Cicolini G (2017). Relationship between peripheral insertion site and catheter-related phlebitis in adult hospitalized patients: a systematic review. Prof Inferm.

[10] Olaechea PM, Palomar M, Alvarez-Lerma F, Otal JJ, Insausti J, Lopez-Pueyo MJ (2013). Morbidity and mortality associated with primary and catheter-related bloodstream infections in critically ill patients. Revista espanola de quimioterapia : publicacion oficial de la Sociedad Espanola de Quimioterapia.

[11] Timsit JF, Bouadma L, Ruckly S, Schwebel C, Garrouste-Orgeas M, Bronchard R (2012). Dressing disruption is a major risk factor for catheter-related infections. Crit Care Med.

[12] O'Grady NP, Alexander M, Burns LA, Dellinger EP, Garland J, Heard SO (2011). Guidelines for the prevention of intravascular catheter-related infections. Clin Infect Dis.

[13] CDC Guidelines for the Prevention of Intravascular Catheter-Related Infections. Update 2017. Available from: https://www.cdc.gov/infectioncontrol/guidelines/bsi/recommendations.html. 2017.

