# Supplementary material for: Lower risk of peripheral venous catheter-related bloodstream infection by hand insertion
Source: Antimicrob Resist Infect Control. 2022 Jun 3;11:80. doi: 10.1186/s13756-022-01117-8 (PMC9164319; doi:10.1186/s13756-022-01117-8)
Supplement: Supplementary file 1 — Additional file 1: Table S1. Patients with peripheral catheters in the upper extremity. Table S2. Univariable Cox model for PVC-related BSI. Table S3. Multivariable marginal Cox model for PVC-related BSI. Table S4. Univariable Cox models for PVC-related BSI stratified by different PVC duration. Table S5. Microorganism distribution according to the insertion site. [file 13756_2022_1117_MOESM1_ESM.docx]

**Additional file 1**

**Supplementary Table 1: Patients with peripheral catheters in the upper extremity**

|  |  | n (%) |
| --- | --- | --- |
| Sex* | Female (%) | 73325 (53.9) |
|  | Male (%) | 62644 (46.1) |
| Age*, median [IQR] | | 49 [32 ; 69] |
| Number of PVC per patient stay, mean (SD) | | 1.7 (1.59) |
| ICU department (%) | | 9152 (2.3) |
| Time to catheter insertion, median [IQR] | | 1 [1 ; 4] |
| Catheter days, median [IQR] | | 3 [2 ; 4] |
| Insertion site | Hand (%) | 109686 (27.2) |
|  | Forearm (%) | 196737 (48.8) |
|  | Arm (%) | 9710 (2.4) |
|  | Elbow (%) | 41285 (10.2) |
|  | Wrist (%) | 45788 (11.4) |
| Insertion outside of the hospital (%) |  | 32145 (8) |
| PVC-related BSI | | 61 (0) |

Legend. IQR: Interquartile range. ICU: Intensive care unit. PVC: Peripheral venous catheter. BSI: Bloodstream infection. *135’969 patients.

**Supplementary Table 2: Univariable Cox model for PVC-related BSI**

|  |  | Without PVC-BSI | With PVC-BSI | HR | 95% CI | p-value |
| --- | --- | --- | --- | --- | --- | --- |
| Hand (%) |  | 109680 (27.2) | 6 (9.8) | 0.424 | [0.18-0.99] | 0.046 |
| Sex | Female (%) | 203443 (50.5) | 14 (23) | 0.355 | [0.2-0.64] | 0.0006 |
| Age, median [IQR] | | 63 [42 ; 79] | 67 [57 ; 77] | 0.998 | [0.99-1.01] | 0.63 |
| ICU (%) |  | 9151 (2.3) | 1 (1.6) | 0.643 | [0.09-4.62] | 0.66 |
| Operator | insertion outside hospital (%) | 32145 (8) | 0 (0) |  |  |  |
|  | insertion intra-hospital (%) | 371000 (92) | 61 (100) |  |  |  |

Legend. HR: Hazard ratio. CI: Confidence interval. IQR: Interquartile range. ICU: Intensive care unit. PVC: Peripheral venous catheter. BSI: Bloodstream infection.

**Supplementary Table 3: Multivariable marginal Cox model for PVC-related BSI**

|  | HR | 95% CI |  | p-value |
| --- | --- | --- | --- | --- |
| Hand* | 0.419 | 0.178 | 0.985 | 0.046 |
| Sex (Female) | 0.358 | 0.197 | 0.650 | 0.0007 |
| Age^¥^ | 0.997 | 0.988 | 1.006 | 0.56 |
| Time admission-PVC insertion | 0.992 | 0.977 | 1.007 | 0.29 |

Legend. HR: Hazard ratio. CI: Confidence interval. IQR: Interquartile range. ICU: Intensive care unit. *Proportionality risk assumption for the variable “Hand” was respected. ^¥^The log-linearity for age was not respected; after adjusting for a categorical “age” variable (≥65 years and <65 years), we observed similar results with a decreased risk for hand by HR 0.409 (95% CI 0.174-0.961, p=0.040).

**Supplementary Table 4: Univariable Cox models for PVC-related BSI stratified by different PVC duration.**

| PVC-days | HR | 95% CI | p-value |
| --- | --- | --- | --- |
| <3 days | 0.74 | [0.08-7.12] | 0.80 |
| <4 days | 0.31 | [0.04-2.42] | 0.26 |
| <5 days | 0.18 | [0.02-1.31] | 0.090 |
| <6 days | 0.36 | [0.11-1.16] | 0.088 |
| <7 days | 0.40 | [0.14-1.13] | 0.085 |
| <8 days | 0.39 | [0.14-1.09] | 0.072 |
| <9 days | 0.46 | [0.18-1.16] | 0.0997 |

**Supplementary Table 5: Microorganism distribution according to the insertion site**

|  | Other sites | Hand | p-value* |
| --- | --- | --- | --- |
| *Achromobacter* spp | 1 (1.8) | 0 (0) | 0.90 |
| CoNS or other skin commensals | 28 (50.9) | 4 (66.7) |  |
| *Enterobacter* spp | 4 (7.3) | 0 (0) |  |
| Fungi | 2 (3.6) | 0 (0) |  |
| *Klebsiella* spp | 4 (7.3) | 0 (0) |  |
| MRSA | 3 (5.5) | 0 (0) |  |
| MSSA | 7 (12.7) | 1 (16.7) |  |
| *P. aeruginosa* | 2 (3.6) | 1 (16.7) |  |
| *S. marcescens* | 1 (1.8) | 0 (0) |  |
| *S. paucimobilis* | 1 (1.8) | 0 (0) |  |
| Polymicrobial | 2 (3.6) | 0 (0) |  |

Legend. * Fisher test. CoNS: Coagulase-negative staphylococci. MRSA: Methicillin-resistant *Staphylococcus aureus*. Methicillin-sensitive *Staphylococcus aureus*. Spp: species.
